# Green and Effective Removal of Aqueous Graphene Oxide under UV-Light Irradiation

**DOI:** 10.3390/nano8090654

**Published:** 2018-08-24

**Authors:** Xiaoya Yuan, Dong Peng, Qiuye Jing, Jiawei Niu, Xin Cheng, Zijuan Feng, Xue Wu

**Affiliations:** College of Materials Science and Engineering, Chongqing Jiaotong University, Chongqing 400074, China; pd19931123@163.com (D.P.); m15922871980@163.com (Q.J.); Niujw2018@163.com (J.N.); cx1241513800@163.com (X.C.); fzj616@126.com (Z.F.); snowly199303@163.com(X.W.)

**Keywords:** graphene oxide, removal, UV-light, photoreduction

## Abstract

The potential extensive application of graphene oxide (GO) in various fields results in the possibility of its release into the natural environment with negative impacts on humans and the ecosystem. The UV-induced removal behavior of aqueous GO was evaluated in this study, and the effect of various parameters (including initial GO concentration, initial solution pH and co-existing ions) on removal rate of GO were investigated in detail. The results showed that UV-light induced a maximum removal rate of GO of 99.1% after 32 h irradiation without any additives, and that the photo-induced removal process in all cases fitted well with pseudo-first-order kinetics. Under optimal conditions, GO was completely removed, with initial GO concentrations of 10 mg/L while adjusting solution pH to 3 or adding Ca^2+^-containing salt. The GO and photoreduced graphene oxide (prGO) were characterized using High-resolution Transmission Microscopy (HRTEM), X-ray Photoelectron Spectroscopy (XPS), and Fourier-transform Infrared Spectroscopy (FT-IR). The radical species trapping experiments and Electron Spin Resonance (ESR) tests indicated that self-reduction of GO upon UV-light exposure could be achieved via photogenerated electrons from a GO semiconductor. Further mechanism study showed that the high efficiency of UV-induced GO removal came from UV-induced photoreduction, and pH-induced or cation-induced coagulation. This study provided a green and effective method to remove GO from aqueous solutions.

## 1. Introduction

Graphene oxide (GO), one of the most important derivatives of graphene, has many potential applications owing to its unique structures and outstanding physicochemical properties [1,2,3,4]. Because it has many hydrophilic oxygen-containing functional groups, such as hydroxyl and epoxy on the basal planes and carboxylic and carbonyl groups at the edge of sheets, GO can be well dispersed in an aqueous medium, without adding amphiphilic stabilizers [5,6]. With its increasingly widespread use in the fields of hybrid materials, environmental pollutant removal, sensors, nanoelectronics, batteries, and hydrogen storage, photocatalyst, biomedicine and biotechnology, etc. [7,8,9], GO was inevitably introduced into the aquatic environment. However, recent studies show that GO potentially exhibited biological and ecological toxicity [10,11,12]. For instance, Liao et al. [13] reported that GO particles could cause cytotoxicity in human skin fibroblast cells and red blood cell. Chen et al. [14] revealed that GO adhered to and enveloped the chorion of zebrafish embryos, mainly via hydroxyl group interactions, blocked the pore canals of the chorionic membrane, and caused marked hypoxia and hatching delay. Therefore, it is very urgent to eliminate GO from aqueous solution.

In very recent years, different physical and chemical approaches have been developed to remove GO from aqueous solution. For example, Wang et al. [15] used Ca/Al-LDHs as an absorbent for GO removal from aqueous solutions. The removal percentage of GO reached 95% with 1.0 g/L Ca/Al-LDHs dosage and the coagulation process was controlled by electrostatic interactions and bridging function between oxygenated functional groups on GO sheets and the surface of the prepared samples. Duan et al. [2] reported that the removal efficiency of GO (initial concentration = 10 mg/L) with alum coagulation (Al_2_(SO_4_)_3_·14H_2_O) was 80% with 20 mg/L dosage at neutral solution and double-layer compression, charge neutralization, entrapment in precipitates, and bridging effects played important roles in the removal of GO by alum. Very recently, Yuan et al. [16] used a MgAl-MMO composite to remove GO from aqueous solution via chemical approaches, and exhibited ultrahigh GO adsorption capacity (984.2 mg/g at pH = 2) with low dosage (25 mg/L) and fast GO-eliminating rate (within 5 h). The adsorption process was mainly dominated by the well-known memory effect of MMO and pH-induced aggregation of GO. However, most of the methods used to eliminate aqueous GO suffer low efficiency, because the removal mechanisms involved in many cases are based on the physical or chemical interaction between GO nanosheets and the prepared samples. Notably, the additional chemicals employed in these studies would inevitably cause extra pollution after being released into the aquatic environment.

Recent studies show that many oxygen-containing groups on the surface of GO nanosheets were removed upon exposure to UV-light of the GO solution [17,18,19,20], and the resulting prGO should aggregate from aqueous solution due to enlarged hydrophobic domain of the prGO nanosheets. Photoreduction is considered to be a potential alternative to the chemical reduction of GO because of its simplicity of performance and the inexhaustible solar energy. Herein, we systematically investigated UV-light-induced removal of GO from wastewater without any additives. The effect of different solution chemistry, including initial GO concentration, pH value of initial solution, and co-existing ions on this GO removal process was studied in detail. The mechanism of this photo-induced removal of GO was further elaborately discussed. Compared to those methods mentioned above for eliminating GO from the aquatic environment, the photo-induced GO removal described herein is environmentally benign and of low-cost.

## 2. Materials and Methods

### 2.1. Materials

A GO solution with a mass concentration of 3% was purchased from Chongqing Institute of Green Intelligent Technology, Chinese Academy of Sciences. Prior to use, the GO stock solution was diluted and sonicated to a given concentration for the following experiments. All of the chemicals used in the experiments were of analytical grade, and used without further purification.

### 2.2. Characterization

The surface elemental compositions of GO and prGO were determined by XPS using a Thermo Fisher ESCALAB 250XI (Thermo Fisher Scientific Inc., Waltham, MA, USA) photoelectron spectrometer using monochromatic Al Kα X-ray source (hv = 1486.6 eV). FT-IR spectrum was collected on a Bruker Nicolet Nexus 870 spectrometer (Bruker Inc., Karlsruhe, Germany). The morphology and structure were observed by a HRTEM using FEI Tecnai G2 F20 (FEI Inc., Hillsboro, OR, USA) field-emission transmission electron microscopy at an accelerating voltage of 200 kV. ESR experiments were conducted with a Bruker EPR ELEXSYS 500 spectrometer (Bruker Inc., Karlsruhe, Germany).

### 2.3. Photo-Induced Removal of Aqueous GO

The experiments of GO removal induced by UV-light were performed using BILON-CHX-V photoreactor (Shanghai Bilon Instruments Manufacture Inc., Shanghai, China) with a 500 W high-pressure Mercury lamp with maximum wavelength emission at 365 nm (8.85 mW/cm^2^, Shanghai Jiguang Special Lighting Factory, Shanghai, China) as the ultraviolet light source. In each run, 50 mL aqueous GO solution of different initial concentrations was employed to perform the photo-induced removal experiments. During irradiation, 3 mL of the reaction aliquots were withdrawn at certain time intervals and centrifuged at 1 × 10^4^ rpm for 10 min to separate the aggregate from the solution. The GO concentration in the supernatant was determined by UV-visible spectrophotometer (AOE Instruments Inc., Shanghai, China) at a wavelength of 230 nm [2]. The adsorption intensity of aqueous GO solution at 230 nm obeyed the Beer-Lambert Law at GO concentrations below 0.03 mg/mL (Figure S1) [15]. The absorption coefficient (α) for GO at a wavelength of 230 nm was 81.46429 L/(g∙cm). The removal rate (*R*) of GO was calculated by the following equation:

*R* = (*C*_0_ − *C_t_*)/*C*_0_ × 100% (1)

where *C*_0_ and *C*_t_ represented the concentrations of GO before and after irradiation and *t* was the irradiation time. *R* meant removal rate of aqueous GO.

### 2.4. Radical Species Trapping and ESR Experiments

In radical species trapping experiments, isopropanol (IPA, 1 mM), and triethanolamine (TEOA, 1 mM) were used as the scavengers of •OH and h^+^ to probe the active species in photo-induced reaction, respectively. In ESR experiments, the O_2_^•−^ and •OH species were trapped by the 5,5-dimethyl-1-pyrroline N-oxide (DMPO) [21]. Ten milligrams of the as-prepared samples were dissolved in 0.5 mL of deionized water (•OH) or 0.5 mL of methanol (O_2_^•−^), and then 0.5 mL of DMPO (100 mM) was added followed by ultrasonic dispersion for 5 min, respectively. The h^+^ and e^−^ species were trapped by the 2,2,6,6-tetramethylpiperidine-1-oxyl (TEMPO). Ten milligrams of the as-prepared samples were dissolved in 0.5 mL of deionized water (e^−^) or 0.5 mL of methanol (h^+^), and then 0.5 mL of TEMPO (40 mM) was added followed by ultrasonic dispersion for 5 min, respectively.

## 3. Results and Discussion

### 3.1. UV-Light Induced Removal of Aqueous GO

#### 3.1.1. Effect of Initial Concentration on UV-Light Induced Removal of GO

**Figure 1:**
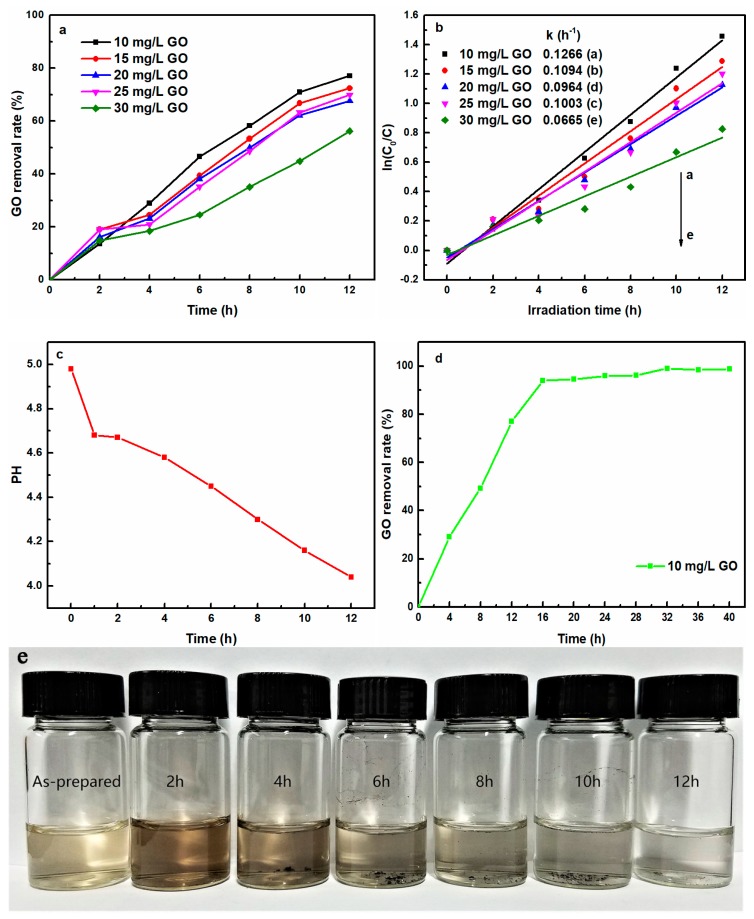
(**a**) Effect of different GO initial concentrations on the UV-light induced removal rate of GO, (**b**) First-order-kinetics-fitted plot for UV-light-induced GO removal; (**c**) Variation of pH value of GO solution with irradiation time; (**d**) Effect of irradiation time on the UV-light induced removal rate of GO (10 mg/L); (**e**) Photographs of evolution of GO solution versus irradiation time (GO = 10 mg/L).

Figure 1a shows the effect of the initial GO concentration (10~30 mg/L) on GO removal rate. Increasing the GO initial concentration reduced the photo-induced removal performance. The UV-light induced removal rates were 77.1%, 72.4%, 67.6%, 69.8%, and 56.2% for initial GO concentration of 10, 15, 20, 25, and 30 mg/L after 12 h irradiation, respectively, indicating that the removal process driven directly only by UV-light was significantly affected by the initial concentration of aqueous GO solution. This phenomenon was attributed to the fact that the GO with a low initial concentration was more easily converted to prGO by photo-initiation.

For a better understanding of the reaction kinetics for the photo-initiated GO removal performance, the experimental data were fitted via a first-order model [22], by ln (*C*_0_/*C*) = *kt*, where *k* represented the rate constant (h^−1^), *C*_0_ and *C* were the initial GO concentrations and at irradiation time *t*, respectively. Figure 1b shows a linear relationship between ln(*C*_0_/*C*) and the irradiation time for GO removal, indicating that the photo-induced removal process in all cases fitted well with pseudo-first-order kinetics. The removal rate constants of different initial concentration of GO were also listed in the inset of Figure 1b. The highest rate constant (0.1266 h^−1^) was found for an initial GO concentration of 10 mg/L, followed by 15, 25, 20 and 30 mg/L. This result clearly indicated that a low initial GO concentration would facilitate its removal under UV light irradiation.

The change of solution pH was also monitored using pH-meter upon exposure of GO solution to UV light. Figure 1c presents the variation of pH value of aqueous GO solution as a function of UV-light irradiation time. The pH value of initial GO concentration was 5.0. Upon UV-light exposure, the GO solution pH gradually shifted to lower. Mozumder et al. [23] reported that H_2_O molecules were able to decompose to produce H^+^ upon UV-light exposure, Equation (2).

(2) $$H_{2}O↔H^{+}+{\mathrm{HO}}^{•}+e_{\mathrm{aq}}^{-}$$

Simultaneously, Ji et al. [24] reported that GO sheets might also contribute to H^+^ when they were reduced. We speculated that the increase in H^+^ concentration resulted from these two processes.

The effect of irradiation time (0–40 h) on the GO removal rate is presented in Figure 1d. Before 16 h irradiation, the UV-light induced removal rate of GO rapidly increased to 93.9% with the prolongation of irradiation time. From 16–32 h irradiation, the removal rate of GO increased slowly, and afterwards remained basically unchanged. The maximum removal rate reached 99.1% at 32 h irradiation, suggesting GO was almost completely removed. This result clearly indicated that the photo-induced process was a green and effective method to remove GO from aqueous solutions.

Figure 1e shows the evolution of the virgin GO solution with the prolongation of the UV-light irradiation time. The color of initial GO suspension slowly changed from light brown to black within 4 h of irradiation and afterwards the supernatant solution became colorless, which was in agreement with a previous report [25]. The fact manifested that the initial hydrophilic GO was gradually photo-reduced upon UV-light exposure, and the hydrophobic prGO aggregated out of the solution. As discussed later in FTIR analysis, due to the photo-initiated reduction, most of the negatively charged oxygen-containing functional groups responsible for the stability of GO in aqueous solution were gradually removed with an increase of the irradiation time, and the hydrophobic regions on the GO nanosheets concomitantly expanded, leading to the sediment of prGO due to the strong interfacial interaction between the graphitic interlayer gallery.

#### 3.1.2. Effect of Initial Solution pH on UV-Light Induced Removal of GO

**Figure 2:**
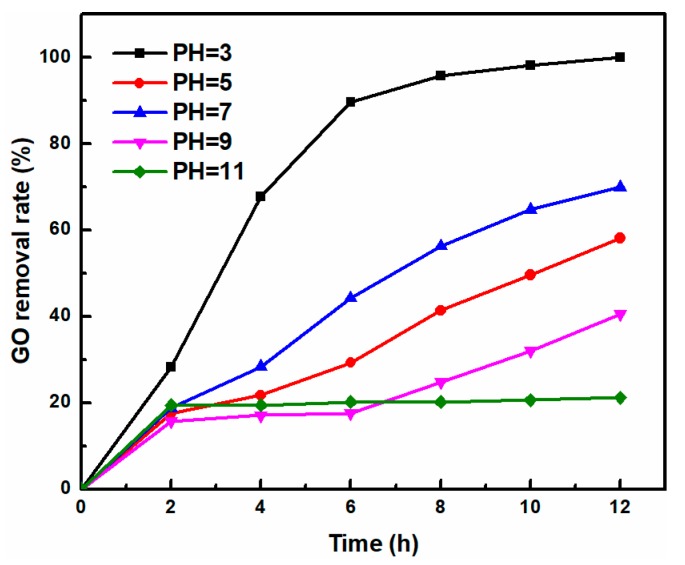
The effect of initial solution pH on GO removal rate (GO = 10 mg/L).

In order to study the effect of solution pH on GO removal by UV-light irradiation, the initial solution pH was adjusted by 0.01 mol/L HCl or NaOH solution and the effect of initial solution pH on UV-light induced removal performance of GO is investigated (Figure 2). At a GO concentration of 10 mg/L, with an initial pH value of GO solution from 3, 5, 7, 9, and 11, the corresponding removal rate was 100%, 58.1%, 70%, 40.5% and 21.2%. Kashyap et al. [26] reported that the pH values have no notable effect on the stability of GO from pH 4.0 to 11.0 in the absence of UV-light. Ji et al. [24] reported that the GO nanosheets in alkaline solution were more easily reduced into prGO upon UV-light irradiation. However, our experimental data showed that increasing the initial solution pH dramatically decreased the removal rate of GO at pH > 7. The alkaline preferred the deprotonation of carboxyl groups on the edge of GO nanosheets, favoring the stability of prGO throughout the photoreduction process in alkaline solution, which affected the real GO residual concentration of the supernatant solution analyzed by UV-visible spectrophotometer [27]. At pH = 7, the oxygen-containing functional groups on the GO nanosheets were continuously removed upon prolonged UV-light irradiation, and the hydrophobic regions were gradually expanded, eventually causing aggregation of prGO. As solution pH further decreased, GO removal rate first decreased, and then increased, indicating that the H^+^ concentration of the solution had great influence on the GO photo-induced removal process. At pH = 5, the removal rate was reduced due to the partial ionization of oxygen-containing functional groups of prGO [28]. As pH values further decreased from 5 to 3, the carboxyl groups located at the edges of GO nanosheets was easily protonated, leading to fast photoreduction reaction toward aqueous GO under acidic solution, and thus, the GO was completely removed within 12 h due to more prGO aggregates [2,6,26,29].

#### 3.1.3. Effect of Co-Existing Cations and Anions on UV-Light Induced Removal of GO

**Figure 3:**
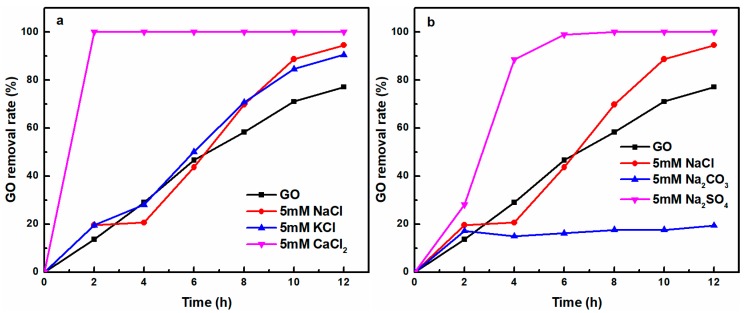
The effect of (**a**) different co-existing cations and (**b**) different anions on the GO removal rate. (GO = 10 mg/L).

To further improve the removal efficiency of GO, the effect of different co-existing cations and anions on the removal rate of GO are investigated (Figure 3). From Figure 3a, GO removal rate increased in the presence of co-existing cations. Very recently, Gao et al. [28] pointed out that the cations could have a destructive effect on the stability of GO to make GO aggregation, the destabilizing ability of cations followed the order of Al^3+^ >> Ca^2+^ > Mg^2+^ > Na^+^ ≈ K^+^. It could be found that the higher the valence state of the cations, the more obvious it was for the coagulation of GO. In our experiment, at the same cation concentration, our results showed that Ca^2+^ ions was more efficient at removing GO than Na^+^ and K^+^ from aqueous solution upon UV-light irradiation, mainly due to the more aggressive binding ability of Ca^2+^ ions with oxygen-containing functional groups of GO; thus, GO was completely removed within 2 h while adding Ca^2+^-containing salt (5 mM) [28]. With a similar binding capacity with oxygen-containing functional groups of GO based on the Schulze-Hardy rule [30,31], Na^+^ and K^+^ showed similar capacity for GO removal. In Figure 3b, the effect of the different anions on removal rate of GO followed the order of Na_2_SO_4_ > NaCl > Na_2_CO_3_. In these experiments, Na^+^ is the counterion. The effect of different anion type on GO removal rate mainly depended on the binding ability of the anion with Na^+^, because the binding ability of them can affect the potency of Na^+^ to destabilize GO [28]. In addition, the increased Na^+^ concentration had a more prominent effect on GO removal than the increased anion concentration [28]. A mole of Na_2_SO_4_ has two moles of Na^+^, while NaCl has only one. Thus, Na_2_SO_4_ exhibits a faster GO removal rate than NaCl due to the electric double layer compression caused by Na^+^. Interestingly, noted that the addition of Na_2_CO_3_ severely suppressed GO removal, which could be ascribed to the good dispensability of prGO in Na_2_CO_3_ alkaline solution.

The GO removal comparison between our UV-light irradiation and other reported methods in the literature is listed in Table 1. It is obvious that the removal percentage of GO described herein was much higher than that of the coagulation employed in several reports. GO may be completely removed from aqueous solution by the photoreduction method with any additional chemicals.

### 3.2. Mechanism of UV-Light Induced GO Removal

#### 3.2.1. Characterization of GO and prGO

**Figure 4:**
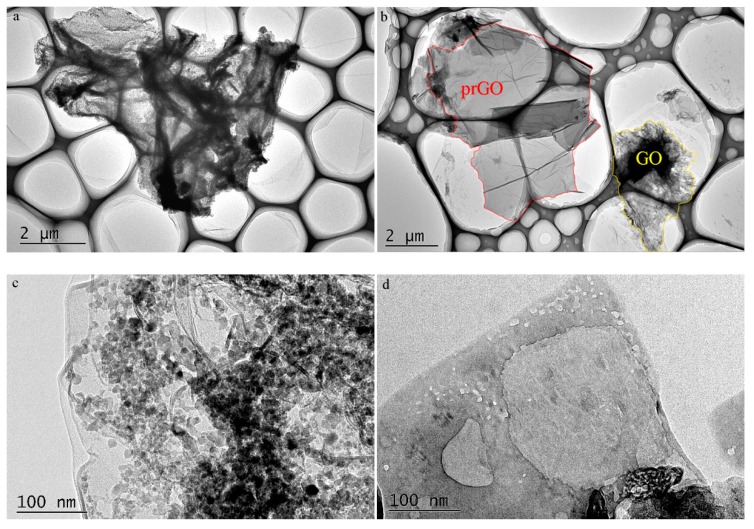
HRTEM images of (**a**,**c**) original GO and (**b**,**d**) the product prGO obtained at the irradiation time of 12 h.

HRTEM was used to directly observe the morphologies of GO and the product prGO obtained at the irradiation time of 12 h. We observed morphological changes before and after the photoreaction. GO has numerous wrinkles and multiple samples clump (Figure 4a). The silk-like aspect of prGO was clearly observed after 12 h photoreduction (Figure 4b), and the prGO exhibited fewer wrinkles and folding, which was also reported in the literature [32,33,34]. Furthermore, compared with GO nanosheets (Figure 4c), the photoreduction resulted in many holes of prGO nanosheets (Figure 4d). So, photoreduction of GO to prGO was successful by UV-light irradiation. The result also indicated that most of the hydrophilic oxygen-containing groups on GO nanosheets were removed upon UV-light exposure of the GO solution. The hydrophobicity of the resulting prGO was increased with prolonged irradiation time, leading to the restacking of the nanosheets. Finally, GO was removed from solution.

**Figure 5:**
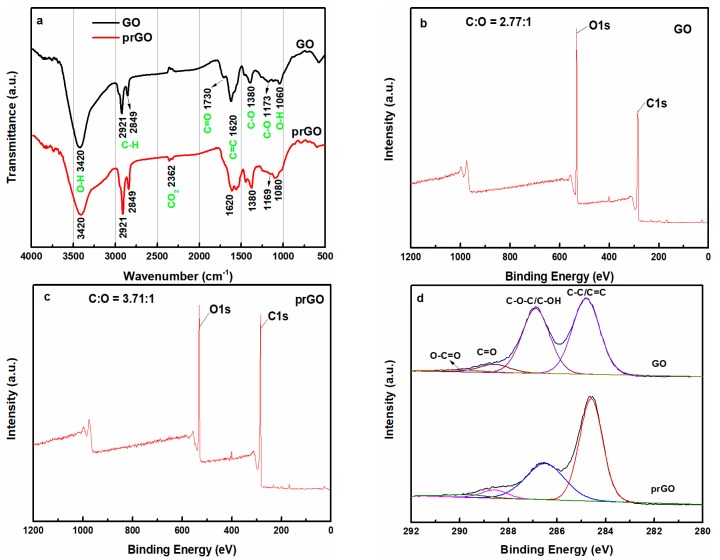
(**a**) FT-IR spectra of GO and the product prGO obtained at the irradiation time of 12 h; (**b**) Survey XPS spectrum of the whole-range spectrum of GO; (**c**) Survey XPS spectrum of the whole-range spectrum of prGO with irradiation time of 12 h; (**d**) C 1s XPS spectra of GO and the product prGO with irradiation time of 12 h.

The photo-induced transformation of GO to prGO was further confirmed by comparing the relative intensity of oxygen-containing groups in the FT-IR and XPS spectra of GO and prGO obtained at the irradiation time of 12 h. As shown in Figure 5a, the FT-IR spectrum of GO exhibited typical absorption bands at 3420, 1730, 1620, 1380, 1173, and 1060 cm^−1^, which may be attributed to O–H stretch, C=O stretch, aromatic C=C, epoxy C–O stretch, alkoxy C–O stretch, and O–H bending, respectively [35,36,37,38]. After 12 h UV treatment, the noticeable decrease in peak intensity at 1730 cm^−1^ implied that a large fraction of the carbonyl group was removed upon UV-light exposure [37]. Furthermore, the peak intensity at 3420 cm^−1^ also decreased, indicating that part of the O–H group had been removed [27]. The survey XPS spectra of Figure 5b,c showed that the C to O atomic ratio increased from 2.77 for GO to 3.71 for prGO, suggesting that oxygenated grouped were removed upon UV light exposure [39]. Figure 5d shows the C 1s XPS spectra of the GO and the product prGO of 12 h irradiation. The peaks at binding energies of 284.6 eV, 286.7 eV, 288.4 eV, and 289.7 eV were assigned to carbon atoms in C–C/C=C, C–O–C/C–OH, C=O and O–C=O, respectively [2,27,40]. After 12 h UV-light irradiation, the peak intensity at 286.7 eV appeared to be weakening notably, revealing that a large fraction of the C–OH/C–O–C groups was removed. The FT-IR and XPS results indicated that the photo-induced removal of GO was successfully achieved via deoxygenation of GO nanosheets, which is in good agreement with the HRTEM analysis.

#### 3.2.2. Mechanism of UV-Light Induced GO Removal

**Figure 6:**
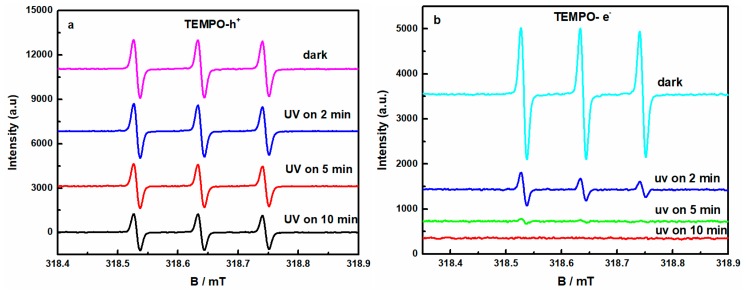

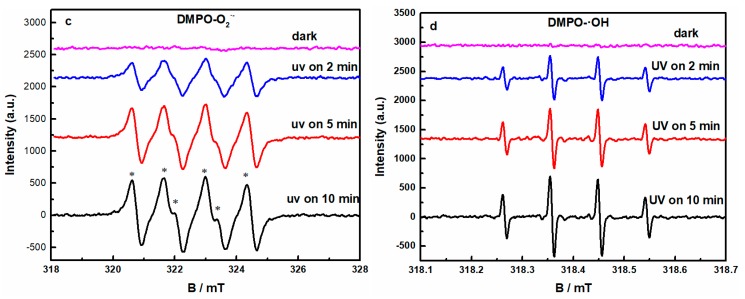
ESR signals of (**a**) TEMPO-h^+^; (**b**) TEMPO-e^−^; (**c**) DMPO-O_2_^•−^; (**d**) DMPO-•OH, for aqueous GO irradiated for 0 min, 2 min, 5 min, 10 min.

The radical species (•OH and h^+^) trapping experiments and ESR tests were executed to investigate the UV-light initiated reaction mechanism involved in our removal of GO. Two explanations have been proposed for the transformation mechanisms of aqueous GO to prGO upon UV-light irradiation. Matsumoto et al. [20] reported that a new photoreaction of GO nanosheets produced H_2_ and CO_2_ by reactions between GO and water under UV irradiation, where a photoprocess similar to that of semiconducting photocatalyst in the mechanism occurred. Simultaneously, Gengler et al. [19] discovered that the ultraviolet light (4.6 eV) was absorbed by the solvent through a nonlinear process, namely two-photon absorption. This process excited the water above its photoionization threshold (6.5 eV), leading to the generation of solvated electrons, which was then responsible for the production of prGO. The ESR tests were carried out to investigate the possible active species (h^+^, e^−^, •OH, O_2_^•−^ and etc.) generated in the reaction systems upon exposure of aqueous GO to UV-light. Figure 6a shows the ESR signals of TEMPO. Three characteristic peaks of the TEMPO could be observed at different time and the peaks intensity gradually decreased with a prolongation of irradiation time, due to the combination of h^+^ species and TEMPO forming the TEMPO-h^+^ adducts to attenuate the ESR signals of TEMPO, which indicated that the h^+^ species were generated in the photo-induced GO removal process. From Figure 6b, three characteristic peaks of the TEMPO could be observed before 2 min UV irradiation; the peaks intensity gradually fade away with a prolongation of irradiation time due to the combination of e^−^ species and TEMPO forming the TEMPO- e^−^ adducts to attenuate the ESR signals of TEMPO, which indicated that e^−^ species were produced in the photoreduction reaction. The holes h^+^ and electrons e^−^ are produced by π–π* band UV excitation in the π-conjugated domains of GO, Equation (3) [17,41]. Figure 6c shows the ESR signals of DMPO-O_2_^•−^ for the GO [21,42]. There was no peak in the dark, only four characteristic peaks of the DMPO-O_2_^•−^ adducts appeared, and the intensity gradually enhanced with a prolongation of irradiation time. Furthermore, the other two characteristic peaks of the DMPO-O_2_^•−^ adducts appeared up to 10 min UV irradiation, and the peaks’ intensity were very weak, which revealed that few O_2_^•−^ species existed in the photo-initiated reaction. The O_2_^•−^ were yielded because the ubiquitous oxygen molecules at the GO surface trapped the e^−^, and finally, the O_2_^•−^ converted into •OH, Equation (4) [43,44]. From Figure 6d, there was no peak in the dark, four characteristic peaks of the DMPO–•OH adducts could be observed and the peaks intensity gradually increased with a prolongation of irradiation time, which revealed that •OH species were produced in the photoreduction reaction process [21,42]. Part of the •OH was from the conversion of O_2_^•−^, and another part due to trapping of the h^+^ by OH^−^ groups or by H_2_O to produce, Equation (5) [45]. The ESR results indicated that GO as a semiconductor was able to generate electron-hole pairs under UV-light irradiation. We believe that these electrons reduced GO to prGO, Equation (6) [20]. Therefore, a possible mechanism of UV-induced removal of GO is the self-reduction of the GO semiconductor under UV-light irradiation.

(3) $$\mathrm{GO}\overset{\mathrm{hv}}{\to }\mathrm{GO}\left(h_{\mathrm{vb}}^{+}\right)+e_{\mathrm{aq}}^{-}$$

(4) $$O_{2}+e_{\mathrm{aq}}^{-}\to {O_{2}}^{•-}\overset{H^{+}}{\to }•{\mathrm{HO}}_{2}\overset{H^{+}+e_{\mathrm{aq}}^{-}}{\to }H_{2}O_{2}\overset{e_{\mathrm{aq}}^{-}}{\to }•\mathrm{OH}+{\mathrm{OH}}^{-}$$

(5) $$h_{\mathrm{vb}}^{+}+{\mathrm{OH}}^{-}\left({\mathrm{or}\;H}_{2}O\right)\to •\mathrm{OH}\left(+H^{+}\right)$$

(6) $$C-O-C\left(\mathrm{GO}\right)+2H^{+}+2e^{-}\to C-C\left(\mathrm{defect}\mathrm{carbon}\right)+H_{2}O$$

**Figure 7:**
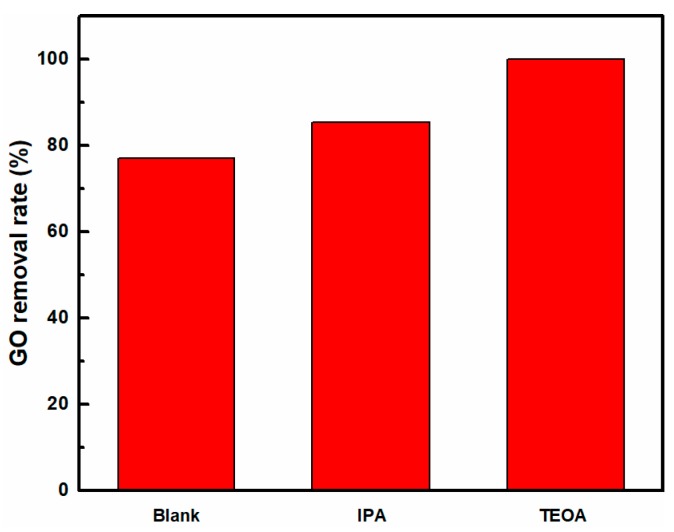
The effect of different scavengers on UV-induced removal rate of GO.

The radical species (•OH and h^+^) trapping experiments were used to further confirm the possible mechanism of UV-induced removal of GO. Different scavengers were used in the UV-induced removal of GO process to quench specific reactive species. The scavengers used in this study were IPA for •OH scavenger, and TEOA to quench h^+^ [21]. The effect of different scavengers on the UV-induced removal rate of GO is shown in the Figure 7. It was observed that the addition of IPA and TEOA made the removal rate of GO increase from 77.1 to 85.3 and 100%, respectively. The results showed that •OH and h^+^ radicals were formed in the photo-induced GO removal system. By promoting the positive reaction to consume more h^+^, Equation (5), both IPA-trapped •OH and TEOA-trapped h^+^ decreased the recombination of electron-hole pairs so that there were more electrons to participate photoreduction reaction, finally leading to increased removal of GO, which further indicated that the e^−^ produced by exciting GO bandgap were the main active species in this UV-induced GO removal process.

#### 3.2.3. Mechanism of UV-Light and pH-Induced GO Removal

Hu et al. [46] reported that at low pH values, the groups (carboxylic and/or hydroxyl group) on GO were protonated, rendering the decrease of the surface charge density which caused GO coagulation. As discussed in Part 3.1.2, the highest removal rate of GO was obtained at pH = 3. To investigate the pH-induced removal of GO, the GO solution (10 mg/L) was adjusted to pH = 3 and stirred for 12 h without turning on the light; the pH-induced GO removal rate was then calculated. In fact, the pH-induced GO coagulation capacity was about 19% of that upon UV-light exposure at pH = 3, indicating that the driving force of GO removal at pH = 3 was not primarily pH-induced coagulation under UV-light irradiation. Thus, the mechanism of the GO removal using UV-light irradiation was the self-reduction of GO and pH-induced coagulation of GO. This self-reduction of GO under UV-light irradiation was predominant in determining the removal rate of GO in aqueous solution. At low pH values, pH-induced coagulation of GO could play a minor part in removing GO from the solution.

#### 3.2.4. Mechanism of UV-Light and Cation-Induced GO Removal

Adding cations to aqueous GO solutions could enhance GO removal efficiency because the cations were able to induce GO coagulation. To investigate the cation-induced removal of GO, different cations salts (5 mM CaCl_2_, 5 mM NaCl and 5 mM KCl) were added to GO solution (10 mg/L) and stirred for 12 h without light irradiation; the cation-induced GO removal rate was then calculated. In fact, the Ca^2+^, Na^+^ and K^+^ -induced GO coagulation capacities were about 100%, 23.6%, 23.9% of that upon simultaneous UV-light exposure, respectively. The result indicated that the Ca^2+^ -induced coagulation of GO under UV-light irradiation was predominant in determining the removal of GO from aqueous solution. Multivalent cations (Ca^2+^) were reported to hold high binding capacities with oxygen-containing functional groups of GO, and easily penetrate the electric double layer to aggregate GO via complexing [28]. In contrast, the Na^+^ and K^+^ induced GO aggregation was established mainly by the electric double-layer suppression [28]. The data from these control experiments indicated that the driving force of GO removal in the presence of monovalent cations was not mainly from cation-induced coagulation under UV-light irradiation. That is to say that the possible mechanism of the UV-light-induced GO removal in the presence of co-existing cations was the self-reduction of GO and cation-induced coagulation of GO. For co-existing multivalent cations, light-induced GO removal could be mainly driven by the cation-induced coagulation; otherwise, the self-reduction of GO under UV-light irradiation was predominant in eliminating GO from aqueous solution.

## 4. Conclusions

In summary, UV-light removal of GO from aqueous solution was successfully achieved. The UV-induced removal rate of GO was dependent on the initial GO concentration, the initial solution pH, and co-existing ions. The UV-induced removal performance of GO could proceed efficiently by adjusting initial solution pH and adding co-existing multivalent cations. Highly efficient GO removal behavior came from UV-induced photoreduction, and pH-induced or cation-induced coagulation. Direct UV-light irradiation without any catalysts or additional chemicals is a promising and green method to remove GO from aqueous solutions.

## Figures and Tables

**Table 1:** The removal performance of GO by UV-light irradiation as compared with other methods.

Methods	Materials(Dosage)	pH	Initial Concentration of GO	Removal Rate (%)	References
Photoreduction	–	7	10 mg/L	99.1%	This study
	–	3		100%	
	Ca^2+^ (5 mM)	7		100%	
Coagulation	Mg/Al-CO_3_-LDH (1.0 g/L)	7	60 mg/L	70%	[7]
	Mg/Al-Cl_3_-LDH (1.0 g/L)	7		95%	
Coagulation	Ca/Al-LDHs (1.0 g/L)	7	15 mg/L	93.8%	[15]
	Ca/Al-LDHs (1.0 g/L)	7		88.7%	
Coagulation	Al_2_(SO_4_)_3_·14H_2_O (20 mg/L)	7	10 mg/L	80%	[2]

## Supplementary Materials

The following are available online at http://www.mdpi.com/2079-4991/8/9/654/s1, Figure S1: The correlation between GO concentration and its UV absorbance intensity at 300 nm.
