# Supplementary material for: Green and Effective Removal of Aqueous Graphene Oxide under UV-Light Irradiation
Source: Nanomaterials (Basel). 2018 Aug 24;8(9):654. doi: 10.3390/nano8090654 (PMC6164349; doi:10.3390/nano8090654)
Supplement: Supplementary file 1 [file nanomaterials-08-00654-s001.pdf]

Supplementary Information

Green and Effective Removal of Aqueous Graphene Oxide under UV-Light Irradiation

Xiaoya Yuan *, **Dong Peng, Qiuye Jing, Jiawei Niu, Xin Cheng, Zijuan Feng** and **Xue Wu**

College of Materials Science and Engineering, Chongqing Jiaotong University, Chongqing 400074, China; pd19931123@163.com (D.P.); m15922871980@163.com (Q.J.); Niujw2018@163.com (J.N.); cx1241513800@163.com (X.C.); fzj616@126.com (Z.F.); snowly199303@163.com(X.W.)

* Correspondence: yuanxy@cqjtu.edu.cn (X.Y.); Tel./Fax: +86-23-62789154

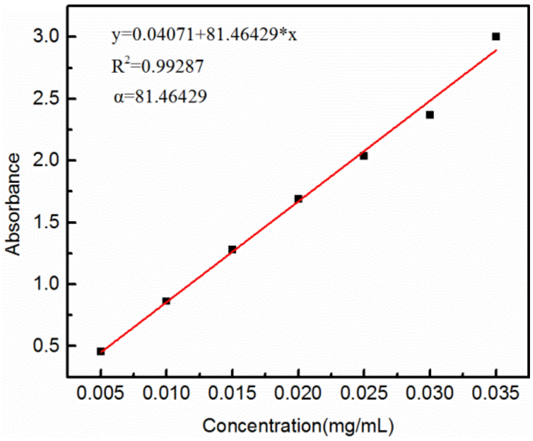

Figure S1. The correlation between GO concentration and its UV absorbance intensity at 300 nm.
